# Essential agriculture, sacrificial labor, and the COVID‐19 pandemic in the US South

**DOI:** 10.1111/joac.12522

**Published:** 2022-10-24

**Authors:** Caroline Keegan

**Affiliations:** ^1^ Department of Geography Texas A&M University College Station TX USA

**Keywords:** agricultural exceptionalism, COVID‐19, essential workers, farm labor, racial capitalism, US South

## Abstract

As farmworkers were reframed as “essential” workers during the COVID‐19 pandemic, US growers demanded unfettered access to foreign farm labor. After initially announcing a freeze on all immigration processing, the Trump administration bowed to farmers' demands, granting a single exception for agricultural guestworkers under the H‐2A visa program. Through a focus on H‐2A farmworkers in Georgia, this paper highlights how the pandemic exacerbated farm labor conditions in the US South. The author interrogates these conditions through the lens of racial capitalism, exposing the legacies of plantation political economies and a longstanding agricultural labor system premised on devaluing racialized labor. These histories are obscured by the myth of agricultural exceptionalism—the idea that agriculture is too different and important to be subject to the same rules and regulations as other industries. Agricultural exceptionalism naturalizes the racial capitalist system and informs state responses that privilege agricultural production *through* the exploitation of farmworkers, remaking “essential” farmworkers as sacrificial labor.

## 1 INTRODUCTION

As the COVID‐19 pandemic spread across the US in March 2020, the Trump administration announced a halt on all immigration processing. There was immediate backlash from farmers in the United States, who argued that without temporary foreign agricultural guestworkers through the H‐2A visa program, crops would rot in the fields and Americans would go hungry. Georgia's then‐US Senators David Perdue and Kelly Loeffler signed onto a letter with several other members of Congress asking that special exceptions be made for H‐2A visas. The letter explains: “Food security is national security. We believe suspending visa services that our farmers rely on will be detrimental to families across our nation trying to put food on the table” (quoted in Williams, 2020). Georgia Governor Brian Kemp also spoke out in defense of Georgia farmers, affirming his “unwavering support for Georgia's oldest and largest industry,” stating, “we will continue to fight for Georgia's agriculture industry and the families who feed and clothe the world from right here in the Peach State” (quoted in Kneiser, 2020). Politicians and media praised the patriotism, hard work, and ingenuity of US farmers braving the pandemic crisis (Ethridge, 2020; Kennedy, 2020). In response, the Trump administration quickly backpedaled, announcing in April 2020 that a special exception would be made only for H‐2A visas and that, in fact, the H‐2A visa process would be streamlined by easing some of the application requirements for farmers (Weise, 2020).

The H‐2A agricultural guestworker visa program, which has its roots in World War II‐era foreign labor importation schemes and was officially codified in 1986, provides short‐term legal immigration status to foreign farmworkers, the vast majority of whom, currently, are men from Mexico and Central America (Fleischer et al., 2013). Employers who apply for and “import” H‐2A workers are required to provide housing and transportation to workers, making H‐2A workers uniquely dependent on their employer for far more than their wages. H‐2A workers are tied to a single employer through their visa, making them unable to change jobs if working conditions are poor. Abuses like wage theft, substandard housing, illegal fees, and retaliation against whistleblowers are common (Silva & McCausland, 2021). While H‐2A workers may spend the majority of the year in the United States on an agricultural contract—and in some cases, up to three years—they are nevertheless considered temporary foreign workers, and their employment provides no path to citizenship or legal long‐term residency. Once their contract ends, H‐2A workers are returned to their home country.

Georgia is a national leader in the use of the H‐2A program (US Department of Labor). Georgia growers' preference for H‐2A workers is, in part, informed by Georgia's 2011 anti‐immigrant legislation, HB87, which led to widespread labor shortages in agriculture, with resulting financial losses estimated in the billions of dollars (Black, 2012). Thus, the H‐2A program provides a labor solution to growers in which farmworkers are imported on demand and easily deportable once their usefulness has ended. While growers and farm labor contractors complain about the cost and red tape associated with the program, it nevertheless offers a readily available option to Georgia farmers who oppose both immigration reform and undocumented immigration and yet rely on immigrant labor (Black, 2012). This system is neither natural nor entirely new, but rather evolved out of longstanding agricultural labor relations premised on the exploitation of racialized farmworkers in support of agricultural production.

In times of “crisis,” this uneven labor system intensifies, as production costs and ecological uncertainties, such as drought or late freeze, are reconfigured as downward pressure on farmworkers. During the COVID‐19 pandemic, as H‐2A workers were reframed as “essential” workers—not only to our food system but to our national security—this moniker did not result in higher pay or greater benefits in recognition of their essential status. In fact, in April 2020, then‐US Agriculture Secretary (and former Governor of Georgia) Sonny Perdue announced that he was working “to see how to reduce wage rates for foreign guest workers on American farms, in order to help US farmers struggling during the coronavirus” (Ordoñez, 2020). While, as yet not implemented, these efforts highlight a system by which US farmers are supported by the state *through* the exploitation of farmworkers who already experience a range of economic, socio‐legal, and health vulnerabilities.

The COVID‐19 pandemic illuminates some structural features of the US agricultural system. Agricultural production is framed as food security and *national* security by politicians, media, and farmers, but also as a volatile and vulnerable industry, requiring constant state support. Immigrant farmworkers, when they are considered at all, are framed as “essential” components of the labor supply necessary to “save the crops” (Mitchell, 2012) but are treated as individually disposable and systematically excluded from rural farm communities. While US agricultural policy during the COVID‐19 pandemic focused on supporting the farm “families who feed and clothe the world” (see Kneiser, 2020), farmworkers were overlooked, even as the virus proliferated in cramped farm labor housing. The spread of the disease among farmworkers was so severe that the NGO, Doctors without Borders, was dispatched in the US for the first time in the history of the organization, to a farmworker community in Immokalee, Florida (Mazzei, 2020).

In Georgia, and the US South broadly, the agricultural system is also colored by its plantation past and the durability of devalued, racialized farm labor schemes. Contemporary conditions are shaped by histories of settler colonialism and early racial capitalism—a concept which recognizes the dual, interwoven processes of capitalist development and racial domination (see Robinson, 2000). US agricultural production has always intersected with whiteness, property, and the ideal settler subject. While the particular configurations of racial capitalism have evolved over time, the underlying logic of capital accumulation through racialized exploitation persists. The state‐sponsored system of chattel slavery, which formed the basis of agricultural development in Georgia, was replaced by sharecropping, tenant labor, debt peonage schemes, prisoners‐of‐war (POWs), women and schoolchildren, Caribbean and Latinx migrants, and now, the H‐2A temporary agricultural visa system. Crises like COVID‐19 only exacerbate uneven labor conditions by exposing the sacrificial nature of “essential” farm labor.

In this paper, the author interrogates the conditions for H‐2A workers in Georgia during the COVID‐19 pandemic through an analysis of racial capitalism and the myth of agricultural exceptionalism. Racial capitalism recognizes the historical and systemic interplays of racial domination and capitalist development as a dual process “developed in agriculture, improved by enclosure in the Old World, and captive land and labor in the Americas, perfected in slavery's time–motion field‐factory choreography” (Gilmore, 2017: 225–226). Agricultural exceptionalism refers to the dominant idea in the US that agriculture is too different, vulnerable, and important to be subject to the same laws and policies as other industries (see Thomas, 1986). Despite the realities of modern agribusiness and plantation political economies of the past, agriculture retains a fictional precapitalist character and nostalgic reverence, which function to justify its exceptional status. By tracing the historical development of agricultural exceptionalism and its current effects on Georgia's H‐2A workers during the COVID‐19 pandemic, this paper aims to shed light on an uneven labor system and the ways in which downward pressures on farm labor are mobilized during times of “crisis.” For Georgia's H‐2A workers, physically and socially isolated in rural areas, the racial capitalist system plays out in embodied processes of inclusion and exclusion, regulation and enforcement, access, mobility, safety, and work.

### 1.1 Agricultural exceptionalism, racial capitalism, and Georgia's farm labor system

The myth of agricultural exceptionalism revolves around a nostalgic notion that agriculture is culturally unique and outside of “capitalist logics of accumulation” (Getz et al., 2008: 489). Proponents of the myth argue that agriculture is too different, too vital, and too volatile to be subject to the same laws, policies, and expectations as other industries. Such a framing is not only incompatible with the current reality of industrialized large‐scale agriculture, but also obscures the histories of settler colonialism and plantation political economies which shaped agricultural development in the US (Hofstadter, 1956). Despite its fictional character, the myth of agricultural exceptionalism upholds the figure of the noble family farmer, working to tame the land against the odds. The ideal of the hardworking (white) farmer‐settler functions to justify economic, labor, and immigration policies in support of agricultural production. Yet the figure of the noble farmer is necessarily distinct from the image of anonymous brown bodies working in the fields to harvest crops. In this equation, the noble farmer must be protected from risk and volatility, in part *through* the domination of labor, which is reduced to a farm input. This calculation is codified by state projects and informed by foundational debates about “American” identity, property, and race.

Many scholars tie the myth of agricultural exceptionalism in the US to the New Deal era in which transformative labor laws like the National Labor Relations Act of 1935 and the Fair Labor Standards Act of 1938 contained notable exemptions for farmworkers and domestic workers, both predominantly Black occupations at the time (Rodman, 2016; Thomas, 1986). Many of these legal exemptions remain, including the exclusion of farmworkers from overtime pay, child labor laws, and the right to collectively bargain. These exclusions are premised on the supposedly “different” nature of agriculture as an industry and serve to support agriculturalists at the expense of farmworkers (Getz et al., 2008). One can also point to numerous other state supports for agriculture as evidence of its exceptionalism, including direct government subsidies to agricultural producers, government institutions and agencies designed to support farmers and farming, federally subsidized crop insurance, and a range of immigration and labor laws that ensure the desired flow of foreign workers with few rights and protections. The H‐2A program itself can be understood as a form of state‐sponsored agricultural exceptionalism in that the immigration program applies exclusively to seasonal agricultural workers and provides growers a steady source of labor, while offering H‐2A workers no path to long‐term residency.

The author argues that the myth of agricultural exceptionalism dates back further, to the settler‐colonial origins of the colony of Georgia and the foundations of racial capitalism in the US Georgia's Founders championed a paternalistic vision of a territory occupied by “a class of virtuous, diligent white yeoman” (Scott, 1995: 27). Beyond constructing the ideal subject, the founders believed a densely populated farming colony made up of armed white settlers would provide the militia power necessary to defend the territory and support the colonies to the north (Trustees, 1732). For this reason, in 1735, James Edward Oglethorpe, a key founder of the colony of Georgia (and himself an enslaver), spearheaded the unprecedented move of banning slavery in Georgia, not out of humanitarian concern, but rather in defense of white settlement. The “Act for rendering the Colony of Georgia more Defensible by Prohibiting the Importation and use of Black Slaves or Negroes in the same,” explained that the protection of the colony depended on a society of free whites given that “slaves were liable to rebel, especially in time of war” (in Scott, 1995: 27). Thus, the farmer–settler ideal was constructed through and against narratives of threatening, racialized others, a process in which whiteness and property were always linked to racial domination and Indigenous dispossession (see Harris, 1993).

These concerns were soon set aside in response to demands from elite settlers who argued that without slavery the “poor people of Georgia, may as well think of becoming Negroes themselves” (from whose condition at present they seem not to be far removed) (Stephens, 1742). By 1750, chattel slavery was legal in Georgia, and by the “Declaration of Independence” in 1776, half of the population of Georgia consisted of enslaved people in a system of permanent bondage (Scott, 1995). This racial capitalist system was maintained through sanctioned and highly visible violence (Wood, 1987). While the majority of Georgia farmers held few or no slaves, wealthy planters exerted disproportionate power over social, political, and economic life across the US South, establishing plantation political economies (Woods, 2017). Thus, the earliest demands for state‐supported agricultural exceptionalism revolved around the establishment of a system of plantation slavery in which enslaved Africans were held in permanent bondage, a system justified, in part, through arguments that enslaved labor was “essential” to the success of agricultural production and to the very survival of the colonies (Scott, 1995).

In his theory of racial capitalism, Robinson (2000: 187) describes the necessity of framing indigenous and Black peoples as less than human to justify the “destruction of the native savage and the domination of the imported one” necessary for building the “systems of manufacturing, plantation slavery, and farming [of] an integrated national economy sharing the exploitation of land, labor, and natural resources.” The development of the US agricultural system was at the heart of intersecting goals of capitalist development and settler colonialism. These plantation political economies informed an international system of racial capitalism and global finance whereby enslaved Black labor depressed wages beyond agricultural industries and investments in plantation industries (cotton, tobacco, and mortgages on enslaved people themselves) spurred financial speculation and economic growth beyond the South. As Du Bois (1935/1998, p. 5) argues: “Black labor became the foundation stone not only of the Southern social structure, but of Northern manufacture and commerce, of the English factory system, of European commerce, of buying and selling on a world‐wide scale.”

Examining the US agricultural labor system through the lens of racial capitalism exposes the linkages through history with regard to the strategic devaluing of racialized labor. As Pulido argues, “[b]y insisting that we are still living with the legacy of these processes, racial capitalism requires that we place contemporary forms of racial inequality in a materialist, ideological, and historical framework” (2017: 527). In Georgia, the brutal system of chattel slavery that drove plantation political economies and transformed global capitalism retained traces in sharecropping and debt peonage schemes that followed Emancipation (Daniel, 1972; LeFlouria, 2011) and in successive waves of “cheap” labor solutions: POWs, women, schoolchildren, Caribbean and Latinx migrants, and now, H‐2A workers (Okie, 2016).

Through the management of foreign migrant labor, the state plays an explicit role in supporting the “exceptional” nature of agriculture (Burawoy, 1976; Hahamovitch, 2011). In 1917, just months after passing the most restrictive immigration law to date, Congress instituted exceptions only for temporary laborers from the Western Hemisphere and created the first agricultural guestworker program, justified as a national defense policy despite continuing for several years after World War I (Rodman, 2016). Growers' insistence on a surplus labor pool of Mexican farmworkers continued through the 1920s and 1930s despite high domestic unemployment during the Great Depression (Weise, 2020). During WWII, the US once again created agricultural guestworker programs, with the Bracero program, in operation from 1942 to 1964, and the emergency labor importation program (also called the Caribbean program), which evolved into the H‐2 program, and then into the H‐2A program (see Hahamovitch, 2011). In Georgia, growers also accessed European POWs during WWII, who provided a limited supply of cheap wartime farm labor, in addition to Black workers, women, and schoolchildren (Okie, 2016). At each moment of supposed labor shortage, growers seized on the “crisis” to insist on access to a particular *type* of farmworker: foreign, mobile, and individually “disposable”—and the state responded.

Established in 1986, the H‐2A program was relatively small prior to the immigration crackdowns of the early 2000s. Around 2006, it began to increase sharply, surpassing 200,000 H‐2A workers in 2017 (American Farm Bureau, 2017; US Department of Labor, 2019). While H‐2A workers comprise only about 10% of the national agricultural labor force (the majority of which is undocumented), more than 50% of H‐2A workers work in just five states: Georgia, Florida, California, North Carolina, and Washington, where fruit and vegetable production is highly labor intensive (American Farm Bureau, 2017). In 2018, Georgia's growers led in total numbers of H‐2A workers employed, importing 32,364 H‐2A workers, 13.3% of all H‐2A workers brought into the US that year. Georgia's use of the program has grown exponentially since passing one of the harshest anti‐immigrant laws in the country in 2011, resulting in farm labor shortages and harvest losses (Black, 2012). This tension exposes the extent to which immigrant farmworkers are considered absolutely critical—“essential”—to Georgia's agricultural system at the same time that they are criminalized as deviant, “illegal” subjects, or excluded and isolated as temporary foreign “others.” The H‐2A program provides a labor “solution” by which farmworkers are necessarily present and yet “outside” of US identity, rights, and privileges. They are workers but not really residents—and certainly not citizens. They may spend the majority of the year working in the US, but they are defined by their temporary, seasonal, outsider status.

Through agricultural guestworker programs like the H‐2A program, the state facilitates an agricultural system reliant on devalued, racialized labor in which individual workers can be treated as disposable. In the process, the notion of the “disposable” worker (Wright, 2006) appears as a real assessment of lack of skill and social value, rather than the result of dehumanizing forms of labor (see also Peck, 1996: 71). Authorized guestworkers, undocumented workers, and farmworkers with long‐term or permanent legal status all encounter different but interrelated vulnerabilities in the agricultural system. In the US South, they work within a system shaped by plantation political economies premised on racial domination and devalued labor. This labor system has been reproduced through the process of seeking out new groups of exploitable workers in the post‐Emancipation era and continuing into the present. These uneven labor relations exploit hierarchies of race, ethnicity, and citizenship status in the interest of maximizing agricultural profits. This is not the product of a “natural” development in precapitalist agricultural production but an ongoing racial capitalist project informed by a series of actions taken by growers and the state to privilege agricultural production *through* controlling and managing racialized farm labor (Hahamovitch, 1997; Weber, 2015).

### 1.2 Agricultural exceptionalism mobilized during the COVID‐19 pandemic

The historical development of an “exceptional” agricultural system dependent on ever‐evolving racial hierarchies to maintain “cheap” labor has always involved embodied impacts on racialized farmworkers, consequences which go largely unnoticed and uncorrected. Hahamovitch (1997:11) argues that “farm employers … have relied increasingly on people of color to keep their labor costs low and conditions on their farms out of the public limelight. They have averted criticism and conflict by importing workers who can be exploited without apology or protest.” Likewise, Walker (2004: 66) describes the US farm labor system as a “vast, repetitive cycle of recruitment, employment, exploitation, and expulsion” or what Henderson (1999: 83) refers to as “The Ever‐New, Ever‐Same.”

H‐2A workers represent the latest iteration of this trend, providing a source of exploitable, deportable labor that is ever‐present but always “temporary” and “foreign”—somehow both inside and outside of Georgia's agricultural communities. The exclusion of H‐2A workers has a direct relationship with their economic exploitation, social isolation, and adverse health impacts, particularly during the global public health crisis of COVID‐19. As Guthman (2017: 101–102) points out, the “disposability” of farm labor “has mainly rested on labor surplus,” enabling “capitalism to accelerate the extraction of labor power from life—and, for that matter, to poison bodies that work, because other bodies are always available.” These conditions are exacerbated during times of “crisis,” like the COVID‐19 pandemic, in which downward pressures on farmworkers are further mobilized in support of our “food security and national security.”

While farmers' ability to access foreign labor was hotly debated in the early months of the pandemic, framed as an issue of national importance, there were few conversations about the effects of COVID‐19 for farmworkers who are housed in tight quarters, transported on crowded buses, and living in isolated rural areas with little access to health care and other resources. One exception, however, is Florida Governor Ron DeSantis who acknowledged the likelihood of the pandemic spreading among farmworkers and stated, “You don't want those folks mixing with the general public if you have an outbreak” (quoted in Mazzei, 2020). To Governor DeSantis, there is a clear delineation between the migrant farm laborers, who harvest the crops, ensuring food security and national security, and the general population—the Floridians worthy of protection. In this sense, farmworkers are “essential” as *sacrificial* labor, serving the greater purpose of protecting farmers and agricultural production, but not themselves essential human beings.

As the initial shock waves from the COVID‐19 pandemic rippled through the US, growers and agricultural boosters quickly rallied to ensure the flow of foreign farm labor would not be interrupted by the global public health crisis. While all other forms of immigration processing were halted, H‐2A visa applications were streamlined. As of June 2020, H‐2A visas had been approved at a pace 15% faster than in 2019 (Weise, 2020). The Homeland Security Department (2020) has extended a “temporary final rule” throughout the pandemic in order to allow for extended stays of H‐2A workers. This rule is not designed for the benefit of H‐2A workers, but rather, “these flexibilities will ensure that agricultural employers have access to the orderly and timely flow of legal foreign workers, thereby protecting the integrity of the nation's food supply chain and decreasing possible reliance on unauthorized aliens” (Homeland Security Department, 2020). Thus, unfettered access to H‐2A workers serves the dual purposes of protecting food and national security and vilifying undocumented farmworkers.

During this time, the Trump administration also began considering ways to reduce H‐2A wage rates. In response to proposed pay cuts for H‐2A workers during the pandemic, a White House official told NPR that they are “considering all policy options during this unprecedented crisis to ensure our great farmers are protected, and President Trump has done and will do everything he can to support their vital mission” (in Ordoñez, 2020). In addition to the simplified H‐2A application process and attempts to lower farmworker pay, the Trump administration provided a $16 billion COVID‐19 subsidy package for farmers earning less than $900,000, or $1.8 million for a farmer and spouse, income rates far beyond the stimulus caps for the general public. Trump's COVID‐19 farm subsidies follow new trade war subsidies introduced in 2018, as well as existing federal support. As Trump stated in April 2020, “The farmers have been paid a fortune already” (quoted in LaFraniere, 2020).

Georgia's farmers were praised throughout the pandemic as heroes for securing our food supply and persevering despite market changes. Though they faced real challenges, they also received financial support and labor concessions. Despite being “essential,” there was no stimulus for H‐2A workers and no unemployment payments for those who could not work due to COVID‐19 or who chose to remain in their home country rather than travel to the US. While farmers were applauded for their “innovation” and “ingenuity” (Ethridge, 2020; Kennedy, 2020), farmworkers who had to adapt to difficult conditions were not attributed with the same skills.

## 2 EMBODIED IMPACTS OF COVID‐19 FOR H‐2A WORKERS IN GEORGIA

Though Georgia's farm labor system has long created precarious conditions for H‐2A workers—wage theft, human trafficking, substandard housing, and unsafe working conditions are all disproportionately common—the COVID‐19 pandemic brings these issues into sharper focus (Silva & McCausland, 2021). H‐2A workers must carefully balance the risks of their labor—including the risk of contracting COVID‐19—with economic needs as seasonal employment accounts for a significant portion of their annual pay. Despite hundreds of thousands of H‐2A workers traveling into the US during the pandemic, no special safety guidelines were established. According to Georgia Legal Services, no COVID worker protection standards were ever implemented for agricultural employers.

Through a year of H‐2A worker outreach alongside Georgia Legal Services Farmworkers Rights Division, the author witnessed how immigration and labor policies, lax health and safety regulations and enforcement, limited mobility and a lack of transportation, and the physical realities of working and living conditions shape the embodied realities of H‐2A workers who are physically and socially isolated in rural Georgia communities, thousands of miles from their families and care networks. Workers reported no oversight or interaction with agencies like the Department of Labor's or the Occupational Safety and Health Administration, despite describing common issues like wage theft or pesticide exposure. During the pandemic, there were reports that worker complaints to the Department of Labor Wage and Hour Division were met with an investigator's call to the employer, rather than an in‐person inspection.

Because H‐2A workers rely on their employers not only for their wages but also for their housing and transportation, H‐2A workers in rural South Georgia counties struggle to access remote healthcare facilities and rely on their employers for safe living and working conditions. However, news reports documented skyrocketing infection rates among South Georgia's farmworkers, COVID‐positive workers quarantined in the same barracks as healthy workers; and crowded buses transporting workers to and from the fields and on weekly shopping trips (Ethridge, 2020). H‐2A workers had little access to testing and relied on their employer to facilitate testing access. When testing in South Georgia increased, positive case numbers increased exponentially, particularly among farmworkers (Ethridge, 2020).

The exceptional character of agriculture as an “essential” industry is foregrounded in the Georgia Department of Public Health's “Farmworker Guidance” webpage, which begins: “Farming is an essential business operation and activity, often supported by the use of migrant and seasonal farmworkers. COVID‐19 can spread rapidly in settings with many people living in close proximity, including living facilities for migrant farmworkers” (Georgia Department of Public Health, 2021). The guidance goes on to encourage social distancing in the fields (“two blueberry bushes,” “every other field row”), on buses (“limit the number of people in the vehicle”), and separating yourself from others if you or they are ill—all things H‐2A workers have very little control over.

In Georgia, farm labor deaths from COVID‐19 were first reported in June 2020 and included the death of a 17‐year‐old female farmworker (National Center for Farmworker Health, 2021). As of April 2021, more than half a million US farmworkers were estimated to have tested positive for COVID‐19, though this figure likely vastly undercounts the true number (National Center for Farmworker Health, 2021). Even less is known about the long‐term effects of COVID or the compounding effects of COVID in combination with other common health concerns for farmworkers. The lack of available and accurate farmworker data is itself a condition of the farm labor system. Disposability and invisibility go hand in hand, reinforcing exploitation. This is especially true in rural South Georgia agricultural communities, where farmworkers are physically isolated in farm labor housing, often on farms themselves, and socially and culturally isolated and excluded.

Beyond the spread of the disease and lost wages, Georgia's farmworkers face other struggles related to the pandemic. For the first time in 25 years, the Farmworker Family Health Program canceled their free summer healthcare pop‐up, which provided basic healthcare and screenings to farmworkers and their families in South Georgia. Georgia Legal Services Farmworker Rights Division halted legal outreach visits to farmworkers across South Georgia during the pandemic, cutting off one of the few ways H‐2A workers could access legal services and know‐your‐rights information. Workers also experienced increased social isolation, struggling to access Spanish‐language information about COVID‐19, working in rural areas with patchy reception, and living thousands of miles from their care networks.

In Georgia, farmworkers were not prioritized at early stages of vaccine access and vaccination rates lag behind the general population (Johnson, 2021). In March 2021, Georgia Legal Services implored Governor Kemp to develop a plan “to ensure that farmworkers in the State of Georgia have fair and equitable access to the COVID‐19 vaccine” (Mercado‐Spencer, 2021). This paper acknowledges the “extra barriers that farmworkers face,” including inaccessible English‐only public health information, a lack of legal documentation (particularly for undocumented workers), and the lack of institutional trust and community building necessary to assure that farmworkers access the information and vaccines they need. Rather than develop a comprehensive plan, however, the state ignored such requests and the burden fell to underfunded community organizations like the Southeast Georgia Communities Project, who organized inoculation drives and food distributions throughout the pandemic.

## 3 CONCLUSION

These are only a few reflections related to the experiences of H‐2A workers in Georgia during the COVID‐19 pandemic, which is still playing out and the full scope of which we may never know. What is clear, however, is that COVID‐19 has exacerbated uneven labor relations, exposing the sacrificial nature of the farmworkers within labor system. These conditions are informed by the myth of agricultural exceptionalism, its state supports, and its historical linkages to US settler colonialism and racial capitalism. The effects of legal exemptions and institutional processes play out in embodied, everyday moments for Georgia's H‐2A workers who live these realities. As burdens are shifted away from agricultural producers, the weight often falls on workers' backs, who are treated as collectively essential but individually disposable. The deadly possibilities of this racial capitalist system increase in clarity amidst this global public health disaster.

During the COVID‐19 pandemic, US farmers have accessed increasing numbers of foreign farmworkers despite pauses in all other forms of immigration processing. Despite broad recognition among government agencies and public health officials that H‐2A workers live and labor in especially risky conditions, the visa program was streamlined, with no specific mandates for protecting foreign workers from COVID‐19 and little to no inspection of safety and health conditions. These policies and exemptions are focused on supporting growers and agricultural production—not farmworkers. Even more explicitly, the Trump Administration attempted to lower wages for “essential” H‐2A workers. This would appear to be a contradiction were it not for an analysis of the historical development of the agricultural system in which “essential” farm labor was *always* sacrificial; farmworkers are collectively indispensable but individually disposable. The H‐2A system represents the latest labor solution in an agricultural system premised on devaluing racialized labor in support of the ideal farmer‐settler and in defense of the idea of industrialized agriculture as “national security.”

But while this system presents itself as a necessity in the name of food security and national security, this racial capitalist configuration is in reality a set of processes continually made and remade. As the COVID‐19 pandemic places H‐2A workers in increasingly risky positions, we must question the logic that privileges agricultural production and capital accumulation through systems of sacrificial labor. By tracing the historical materialist conditions of Georgia's farm labor system, these realities are exposed as merely the latest iteration of political, social, and economic systems steeped in longstanding racialized power relations.

## CONFLICTS OF INTEREST

This work has not been funded by any external sources or grants. The authors have no conflicts of interest to declare.

## Data Availability

The data that support the findings will be available in University of Georgia ProQuest Dissertations Publishing at https://www.proquest.com/pqdtglobal/docview/2572559844/76AC6468BFF44E0DPQ/2?accountid=14537 following an embargo from the date of publication to allow for commercialization of research findings.
